# Effect of dexmedetomidine pretreatment on lung injury following intestinal ischemia-reperfusion

**DOI:** 10.3892/etm.2013.1317

**Published:** 2013-09-26

**Authors:** JINMEI SHEN, GAN FU, LILI JIANG, JUNMEI XU, LI LI, GAN FU

**Affiliations:** 1 Department of Anesthesiology, Second Xiangya Hospital, Central South University, Changsha, Hunan 410011, P.R. China; 2 Department of Hematology, Xiangya Hospital, Central South University, Changsha, Hunan 410008, P.R. China

**Keywords:** intestinal ischemia-reperfusion injury, dexmedetomidine hydrochloride, tumor necrosis factor-α, interleukin-6

## Abstract

Reperfusion injury is tissue damage caused by the re-supply of blood following a period of ischemia in tissues. Intestinal ischemia-reperfusion injury (IRI) is an extremely common clinical event associated with distant organ injury. The intestine serves as the initial organ of multi-system organ dysfunction syndrome. It is extremely important to identify a method to protect against IRI, as it is a key factor associated with morbidity and mortality in patients. In the present study, the protective effects of pretreatment with dexmedetomidine hydrochloride were investigated. Rats were divided into six groups and models of intestinal ischemia were created in the five groups. Certain groups were pretreated with dexmedetomidine hydrochloride. The levels of TNF-α and IL-6 were measured by enzyme-linked immunosorbent assay in order to evaluate the injury. Tissue sections were stained with hematoxylin and eosin to visualize the damage. qPCR and western blotting were performed to examine the inflammatory status. Pretreatment with various doses of dexmedetomidine hydrochloride significantly reduced the pathological scores and the inflammatory reaction. The levels of TNF-α, IL-6, TLR4 and MyD88 were decreased in the dexmedetomidine hydrochloride treatment groups compared with those in the sham control and untreated ischemia reperfusion groups. The results of the present study indicate that pretreatment with dexmedetomidine hydrochloride may be a useful method of reducing the damage caused by IRI.

## Introduction

Interruption of the blood supply to tissues results in rapid changes in the environment of cells. The resulting absence of oxygen and nutrients creates a condition in which the restoration of blood flow causes oxidation and inflammation. Intestinal ischemia-reperfusion injury (IRI) is an important factor associated with high morbidity and mortality in patients (1). Intestinal IRI is associated with the exacerbation of intestinal injury and a systemic inflammatory response leading to progressive distal organ impairment, finally resulting in cardiocirculatory, respiratory, hepatic and renal failure (2,3). Intestinal IRI is also associated with other diseases, including septic hypovolemic shock (4,5). The process involved in intestinal IRI and protective treatment strategies have been studied for a number of years. Several trials have provided successful methods to attenuate the injury effect of intestinal IRI. Ischemic preconditioning, antioxidants, NO supplementation, anticomplement therapy, antileukocyte therapy, perfluorocarbons, enteral feeding, glutamine supplementation and glycine supplementation have been well studied (6).

Dexmedetomidine hydrochloride is an *S*-enantiomer of medetomidine, chemically described as (+)-4-(S)-[1-(2,3-dimethylphenyl)ethyl]-1H-imidazole monohydrochloride. This drug remains the first and the only α_2_ agonist indicated for sedation (7), and is often used by Intensive Care Units and Anesthesiology Departments. Dexmedetomidine hydrochloride has sedative, analgesic, sympatholytic and anxiolytic effects that blunt a number of the cardiovascular responses in the perioperative period. The sedative reduces the requirements for volatile anesthetics, sedatives and analgesics without causing significant respiratory depression (8). Dexmedetomidine hydrochloride may be useful for the deleterious cardiovascular effects of acute cocaine intoxication (9). Dexmedetomidine hydrochloride may also offer a new paradigm in the pharmacological treatment of symptoms of distress at the end of life. Previous studies have shown that dexmedetomidine hydrochloride exhibits a protective effect in a number of tissues with IRI (10–12). Gu *et al*(13) observed that renal IR significantly induced pulmonary injuries, increased the wet/dry (W/D) ratio, enhanced MPO activities and increased ICAM-1 and TNF-α mRNA levels in mice. Pre- and post-treatment with dexmedetomidine was demonstrated to markedly reduce lung edema and inflammatory response and lower MPO activity and ICAM-1 and TNF-α mRNA expression. Kiliç *et al*(14) reported that dexmedetomidine treatment leads to biochemical and histopathological benefits by preventing the IR-associated cellular damage of intestinal and renal tissues, as shown in rabbits. In the current study, the potential effects of dexmedetomidine hydrochloride on lung injury caused by intestinal IRI were investigated in rats.

## Materials and methods

### Animal models and surgery

Male Sprague-Dawley rats (8 weeks-old; 250–270 g; n=36) were provided by the Second Xiangya Hospital of Central South University (Changsha, China). All surgical procedures were performed according to the Regulations for the Administration of Affairs Concerning Experimental Animals (Approved by the State Council on October 31, 1988 and promulgated by Decree No. 2 of the State Science and Technology Commission on November 14, 1988). Rats were acclimatized for one week following transfer to the Department of Anesthesiology, Second Xiangya Hospital. During this period, rats received food and water *ad libitum*. Prior to surgery, rats were fasted for 24 h with free access to water. Rats were injected with 2% pentobarbital sodium (50 mg/kg). During surgery, incandescence was used to maintain the rectal temperature at 37–38°C. All rats were mechanically ventilated with a standard tidal volume ventilation protocol (tidal volume, 10 ml/kg; respiratory rate, 50–60 breaths/min; inspiratory/expiratory ratio, 1:2). The intestine was exteriorized by midline laparotomy and the intestinal IRI was established by occluding the superior mesenteric artery with a microvessel clip for 1 h, followed by 2 h of reperfusion, as described previously (15,16). The study was approved by the Second Xiangya Hospital, Central South University.

### Groups and drug treatment

Thirty-six rats were randomly divided into six groups (n=6/group). The detailed definitions of each group were as follows: Sham, rats received continuous intravenous infusion of normal saline and sham surgical preparation, including isolation of the superior mesenteric artery without occlusion; ischemia-reperfusion (IR), rats received continuous intravenous infusion of normal saline and intestinal IR was induced by clamping the superior mesenteric artery for 1 h followed by declamping (reperfusion) for 2 h; low dose dexmedetomidine hydrochloride (LDH), rats were pretreated with dexmedetomidine hydrochloride in the tail vein at a dose of 2.5 μg/kg/h for 1 h prior to IR; high dose dexmedetomidine hydrochloride (HDH), rats were pretreated with dexmedetomidine hydrochloride in the tail vein at a dose of 5 μg/kg/h for 1 h prior to IR; yohimbine and dexmedetomidine hydrochloride (Y+D), rats were treated with yohimbine at a dose of 1 mg/kg (>15 min) in the tail vein, followed by 5 μg/kg/h dexmedetomidine hydrochloride for 1 h prior to IR; yohimbine only (Y), rats were treated with yohimbine only at a dose of 1 mg/kg (>15 min) in the tail vein prior to IR. Yohimbine is an antagonist of α2 receptors. We used it to block the effect of dexmedetomidine hydrochloride. All the rats were sacrificed by bleeding in the right ventricular after 2 hours after IR

### Parameters

#### Arterial blood gas (ABG)

Following reperfusion in the intestine, the pressures of oxygen and carbon dioxide and the pH were analyzed in the arterial blood from the left ventricle.

#### W/D ratio of the lung

Two hours following reperfusion, the left lung was harvested, cleaned by removing the cover blood and water and then weighed. Next, the sample was incubated at 70°C for 48 h and weighed. The W/D ratio was calculated as the ratio of the wet weight of the lung to the dry weight.

#### Histopathological evaluation

The lung tissues were embedded in paraffin and then sectioned into 5-μm sections. Slides were stained with hematoxylin and eosin and examined under a microscope (TE300; Nikon, Tokyo, Japan).

#### Histopathology scores

Semi-quantitative analysis of lung histopathology was performed by scoring the tissues based on lung edema, infiltration of inflammatory cells, alveolar hemorrhage, hyaline membrane and atelectasis: no lesion, 0; injured area ≤25%, 1; injured area 26–50%, 2; injured area 51–70%, 3; injured area 70–90%, 4; injured area >90%, 5. A total of three fields were randomly selected for each slide and the average was used as the histopathology score.

#### Enzyme-linked immunosorbent assay (ELISA)

After the rats were sacrificed, the trachea was separated at the bifurcation. Below the tracheal cannula, a ‘V’-shaped gap was cut and a sterile 20G trocar was inserted into the left bronchus. The other end was ligated with a 5-ml syringe. Sterile cold graded saline (10 ml) was poured into the left lung (≤3 ml each time) under the pressure of 20 cm H_2_O. The bronchoalveolar lavage fluid (BALF) was collected in a 10-ml sterile dry centrifuge tube (recovery ratio, >75%), placed on ice and then centrifuged at 500 × g for 10 min. The supernatant was collected and the concentrations of TNF-α and IL-6 were measured by ELISA (Bio-Swamp Life Science, Wuhan, China).

#### RNA isolation and qPCR

Lung tissue was harvested 2 h following reperfusion. Total RNA was isolated using the TRIzol reagent and was then reverse transcribed to cDNA. Real-time quantification of genes was performed using three-step qPCR. The following primers were used: TLR4, forward: GAATGAGGACTGGGTGAGAAAC and reverse: ACCAACGGCTCTGGATAAAGT; MyD88, forward: ACC GCATCGAGGAGGACTG and reverse: CTGTGGGAC ACTGCTCTCCA; and β-actin, forward: AGGCCCCTCTGA ACCCTAAG and reverse: CCAGAGGCATACAGGGACAAC. All primers are purchased from BGI (Shenzhen, China). The reaction conditions were as follows: step 1, 95°C for 2 min; step 2, 95°C for 15 sec, 60°C for 30 sec and 72°C for 1 min; repeat step 2 for additional 39 cycles and 72°C for 10 min.

#### Protein extraction and western blotting

Lung tissue (50 mg) from liquid nitrogen was ground into a powder using a mortar. The powder was diluted in 500 μl RIPA lysis buffer plus protease inhibitors for 15 min on ice. The samples were then sonicated for 10 sec followed by a 10-sec rest. This step was repeated three times. Next, 125 μl 5X protein loading buffer was added to the samples and the samples were boiled for 5 min. The samples were then subjected to sodium dodecyl sulfate-polyacrylamide gel electrophoresis (SDS-PAGE) and transferred to polyvinylidene difluoride (PVDF) membranes. Following blocking with 5% BSA, the anti-IκBα, anti-AKT, anti-P-IκBα, anti-P-AKT and anti-β-actin primary antibodies (Santa Cruz Biotechnology, Inc. Santa Cruz, CA, USA) were added and incubation was conducted overnight at 4°C. Secondary antibodies (Sigma St. Louis, MO, USA) were then added to the membranes for 1 h at room temperature after washing with TBST three times. Finally, the membranes were detected using an X-ray film following enhanced chemiluminescence (ECL) reaction.

#### Statistical analysis

All data are presented as mean ± SD (SPSS 17.0; SPSS, Inc., Chicago, IL, USA). All parameters are based on one-way ANOVA analysis and comparison between groups followed by Bonferroni or Dunnett method. P<0.05 was considered to indicate a statistically significant difference.

## Results

### Body weight, ABG and W/D ratio of the lungs

The average body weight of the rats was 260.16±7.60 g (250–270 g). The rats were randomly divided into six groups. The average body weight, pH and pressure of O_2_ and CO_2_ among the groups were not observed to be significantly different (P>0.05; Table I). Lung injury was measured by lung histopathology scores. IR surgery caused an increase in the ratio of W/D and lung histopathology score. As shown in Table I, dexmedetomidine significantly reduced the W/D ratio and the lung injury caused by IRI. Pretreatment with dexmedetomidine prior to IR (LDH and HDH group) was demonstrated to have a protective effect against lung injury.

### Histopathological evaluation

**Figure 1:**
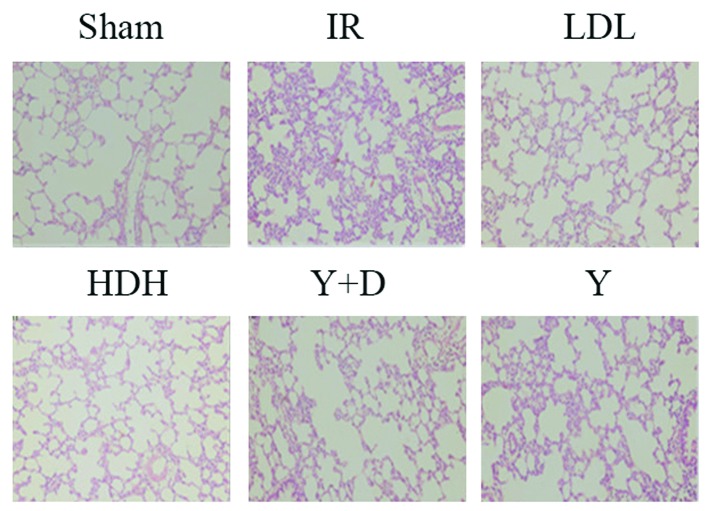
Histopathological evaluation of lungs in various groups. The sections were stained by HE staining; magnification, ×200. IR, ischemia-reperfusion; LDH, low dose dexmedetomidine hydrochloride; HDH, high dose dexmedetomidine hydrochloride; Y+D, yohimbine and dexmedetomidine hydrochloride; Y, yohimbine; HE, hematoxylin and eosin.

Lungs were harvested 2 h following reperfusion, embedded in paraffin and sectioned into 5-μm sections. Slides were stained with hematoxylin and eosin and examined under a microscope (Fig. 1). In the sham group, the integrity of the pulmonary interstitial and alveolar structures was preserved with clear alveolar space and no evident bleeding. The alveolar septa were thick with no edema, and no inflammatory cells had infiltrated into the alveolar septa. In the IR and Y groups, the alveolar structure was severely damaged. The alveolar septa were thick and were infiltrated by a large number of inflammatory cells. A large amount of exudate, red blood cells and inflammatory cells filled the alveolar space. In the LDH group, the alveolar structure exhibited a small amount of damage. A mild edema was observed in the lung interval and exudate was visible as well as a small amount of inflammatory cell infiltration in specific alveolar cavities. In the HDH group, the alveolar structure was rarely destroyed. No thickening or edema was observed in the alveolar septa with little capillary congestion and infiltration of inflammatory cells into the alveolar wall. In the Y+D group, the alveolar structure was partially destroyed with alveolar septal thickening and edema. Parts of the alveolar cavity contained exudate and inflammatory cell infiltration. These observations indicate that pretreatment with dexmedetomidine prior to IR has a protective effect on lung injury, particularly at high doses.

### TNF-α and IL-6 levels in BALF

**Figure 2:**
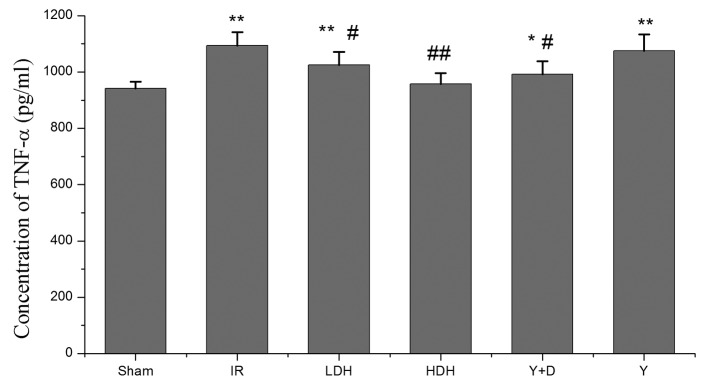
TNF-α concentration in the BALF of lungs among various groups. BALF, bronchoalveolar lavage fluid; IR, ischemia-reperfusion; LDH, low dose dexmedetomidine hydrochloride; HDH, high dose dexmedetomidine hydrochloride; Y+D, yohimbine and dexmedetomidine hydrochloride; Y, yohimbine. ^*^P<0.05 compared with the sham group; ^**^P<0.01 compared with the sham group; ^#^P<0.05 compared with the IR group; ^##^P<0.01 compared with the IR group.

**Figure 3:**
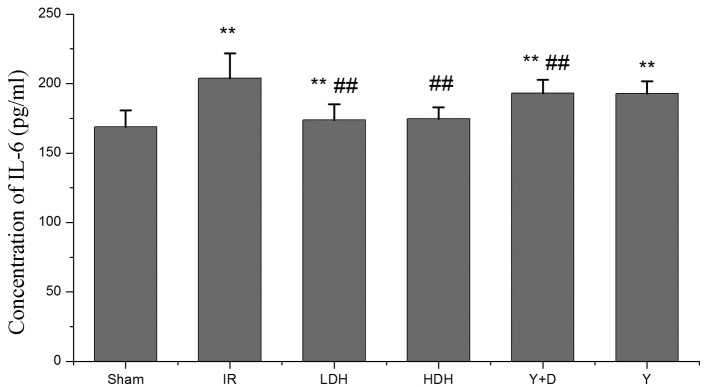
IL-6 concentration in the BALF of lungs among various groups. The same samples as in Fig. 2 were used. BALF, bronchoalveolar lavage fluid; IR, ischemia-reperfusion; LDH, low dose dexmedetomidine hydrochloride; HDH, high dose dexmedetomidine hydrochloride; Y+D, yohimbine and dexmedetomidine hydrochloride; Y, yohimbine. ^*^P<0.05 compared with the sham group; ^**^P<0.01 compared with the sham group; ^#^P<0.05 compared with the IR group; ^##^P<0.01 compared with the IR group.

Intestinal IRI is associated with the exacerbation of intestinal injury and a systemic inflammatory response leading to progressive distal organ impairment. To evaluate the lung injury caused by IRI, BALF was collected following reperfusion and TNF-α and IL-6 levels were examined by ELISA. Four groups (IR, LDH, Y+D and Y) had a higher concentration of TNF-α than the sham group. The differences were statistically significant. The TNF-α levels in the LDH, HDH and Y+D groups were lower than those in the IR group. The HDH group had lower TNF-α levels than the LDH group (Fig. 2). High dose dexmedetomidine hydrochloride appeared to have an stronger effect than a low dose. the IL-6 levels were consistent with the TNF-α levels and showed a similar trend among the groups (Fig. 3). Pro-inflammatory factor production in the lung was considered to indicate the extent of lung injury. The levels of TNF-α and IL-6 among the groups varied, which was consistent with the histopathological evaluation. Therefore, these results indicate that dexmedetomidine hydrochloride pretreatment is a protective method to avoid lung injury by reducing cytokine production.

### TLR4 and MyD88 expression in the lung at 2 h following reperfusion

**Figure 4:**
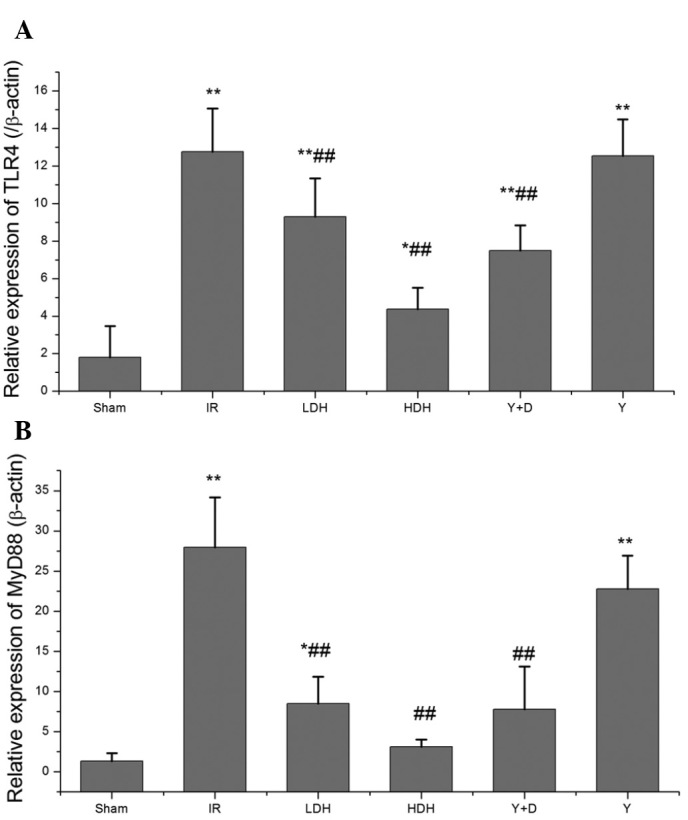
(A) TLR4 and (B) MyD88 mRNA levels in the lungs of various groups. IR, ischemia-reperfusion; LDH, low dose dexmedetomidine hydrochloride; HDH, high dose dexmedetomidine hydrochloride; Y+D, yohimbine and dexmedetomidine hydrochloride; Y, yohimbine. ^*^P<0.05 compared with the sham group; ^**^P<0.01 compared with the sham group; ^#^P<0.05 compared with the IR group; ^##^P<0.01 compared with the IR group.

Since dexmedetomidine hydrochloride was observed to mediate a protective effect against lung injury following intestinal ischemia-reperfusion, molecules upstream of the cytokines were analyzed, including the TLR4/MyD88 pathway which mediates cytokine production. qPCR was performed to determine the levels of TLR4 and MyD88 expression. Compared with their levels in the sham group, the TLR4 and MyD88 levels were increased in the IR, LDH, Y+D and Y groups and the differences were statistically significant (Fig. 4). In addition, the TLR4 and MyD88 levels were decreased in the HDH, LDH and Y+D groups when compared with those in the IR group. This result was consistent with the findings for TNF-α and IL-6 production, indicating that pretreatment with dexmedetomidine hydrochloride may affect the production of TNF-α and IL-6 by altering the expression levels of TLR4 and MyD88.

### IκBα and AKT phosphorylation levels

**Figure 5:**
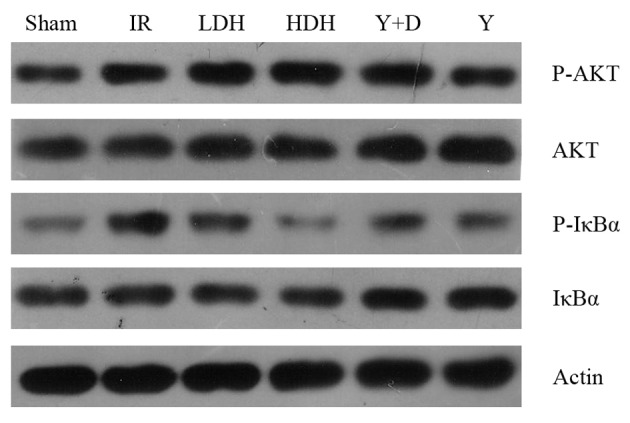
IκBα and AKT phosphorylation levels in the lungs of different groups. IκBα phosphorylation levels were analyzed by western blotting. IR, ischemia-reperfusion; LDH, low dose dexmedetomidine hydrochloride; HDH, high dose dexmedetomidine hydrochloride; Y+D, yohimbine and dexmedetomidine hydrochloride; Y, yohimbine.

NF-κB is an important factor downstream of TLR4/MyD88 and plays a key role in regulating the immune response. Following the observation that TLR4 and MyD88 mRNA levels were reduced in the HDH and LDH groups compared with those in the IR group, western blot analysis of NF-κB activation was performed. IκBα is an inhibitor of NF-κB and blocks its nuclear localization (17,18). Activation of NF-κB is initiated by the signal-induced degradation of IκB proteins. Phosphorylation of IκB is the signal for degradation and therefore, the phosphorylation levels of IκBα were examined (Fig. 5). As the results show, phosphorylation of IκB in the LDH and HDH groups was lower than in the IR group, which was consistent with the mRNA levels of TLR4/MyD88. In addition, the AKT phosphorylation levels were examined, as previous studies have shown that the PI3K/AKT pathway is involved in acute lung injury (19,20). No marked changes in AKT phosphorylation were observed.

## Discussion

IRI of the intestine may cause multi-organ injury and harm to human health. It is associated with a rate of high morbidity and mortality in patients (1). IRI in the intestine results in the production of molecules, including hydrogen peroxide, superoxide and inflammatory cytokines, that may harm distant organs, . This leads to the development of systemic inflammatory response syndrome, which may progress to MOF (21). Intestinal IRI also causes pulmonary infiltration of neutrophils, which contributes to the development of acute respiratory distress syndrome (ARDS) (22,23). Therefore, it is important to reduce the immune response to avoid multi-organ injury. TNF-α release caused by intestinal IRI occurs at the earliest stage of inflammation. It plays a central role in endogenous inflammation to induce endothelial cell injury by promoting IL-1 and IL-6 production. In the acute phase of ARDS, IL-6 promotes inflammatory reaction leading to neutrophil aggregation, infiltration, tissue damage and pulmonary edema (24,25).

Few studies have determined the effect of dexmedetomidine hydrochloride on the immune response. Taniguchi *et al*(26) observed that dexmedetomidine reduced the mortality rate and had an inhibitory effect on the inflammatory response during endotoxemia. The authors identified that endotoxemia, together with dexmedetomidine administration, had an inhibitory effect on hypotension, the production of cytokines and the infiltration of neutrophils into the lungs. Moreover, dexmedetomidine administration, following endotoxin injection, reduced the mortality rate. The inhibitory mechanism of dexmedetomidine on the production of TNF-α and IL-6 remains unclear. There are several studies that have studied the effects of dexmedetomidine and α_2_ agonist-adrenergic receptor agonists on cytokines (27–30). The α_2_ adrenoceptor agonist, paminoclonidine, is known to suppress IL-6 production (27) and clonidine suppresses the production of TNF-α by monocytes (28). α_2_-adrenoceptor agonists have also been observed to modulate LPS-induced TNF-α production by macrophages (29). In a clinical study, dexmedetomidine attenuated IL-6 elevation in post-operative patients (30).

In the current study, dexmedetomidine hydrochloride was shown to have a protective effect on the immune response following IRI in the intestine. Pretreatment with dexmedetomidine hydrochloride significantly reduced the lung injury caused by IRI. In addition, pretreatment with dexmedetomidine hydrochloride prior to IRI reduced IL-6 and TNF-α production in the lung, which was consistent with previous observations of the inhibitory effects of dexmedetomidine hydrochloride on cytokines. To further explore the mechanism, TLR4 and MyD88, upstream factors of IL-6 and TNF-α, were examined. The mRNA levels of TLR4 and MyD88 were decreased, compared with those in the IR group, in the groups pre-treated with dexmedetomidine hydrochloride. In addition, NF-κB activation was attenuated in the dexmedetomidine hydrochloride treatment group, which was consistent with changes in the expression of TLR4 and MyD88. TLR4/MyD88 pathways have been reported to be important for tissue injury following IRI. TLR4- or MyD88-deficient mice exhibit reduced injury levels following IR. Ben *et al*(31) reported that TLR4-deficient mice had significantly reduced lung tissue damage, capillary leakage, lung tissue MPO expression, neutrophil aggregation and reduced TNF-α, IL-6, MCP-1 and MIP-2 mRNA and protein expression levels in lung tissue following intestinal ischemia-reperfusion. Victoni *et al*(32) studied the function of the TLR4/MyD88 signaling pathway in the remote organ acute lung injury caused by intestinal ischemia-reperfusion. The authors observed that MyD88^−/−^ mice had significantly reduced pulmonary vascular permeability leakage, lung tissue MPO expression and neutrophil aggregation and decreased levels of TNF-α and IL-1β expression in the lung tissue, which reduced the mortality rate of the mice (32). The current results in rats also verified the role of TLR4/MyD88 in lung injury due to IRI.

The current study and previous studies indicate that pretreatment with dexmedetomidine may provide useful and effective protection against the harmful effects of IRI. The results also indicate that dexmedetomidine directly suppresses the immune response by modulating the expression of TLR4 and MyD88, which may explain the inhibitory effect of dexmedetomidine on cytokine production. Our results provide a new insight for the clinical use of dexmedetomidine and suggest that a higher dose of dexmedetomidine treatment before ischemic insult produces effective intestinal protection. The effect of dexmedetomidine on other organ injuries caused by IRI requires further investigation.

## Figures and Tables

**Table I:** Weight, ABG and W/D ratio of experiment rats.

Groups	n	Weight (g)	pH	Pressure of O_2_ (mmHg)	Pressure of CO_2_ (mmHg)	W/D ratio	Histopathological score
Sham	6	257.97±8.01	7.41±0.09	153.50±23.87	37.50±4.18	5.71±0.41	2.39±0.83
IR	6	260.53±6.0	7.40±0.07	146.17±18.45	35.67±5.43	7.28±0.50^b^	4.61±0.39^b^
LDH	6	258.02±9.78	7.43±0.09	141.17±29.03	35.50±4.64	6.13±0.82^c^	4.00±0.70^b^
HDH	6	259.93±9.91	7.38±0.07	156.50±34.80	35.33±5.05	5.84±0.53^d^	3.61±0.25^a,c^
Y+D	6	262.38±5.28	7.40±0.07	130.33±13.87	36.50±3.89	6.46±0.62^a,c^	4.12±0.59^b^
Y	6	262.10±7.76	7.37±0.04	137.50±20.01	37.50±5.05	7.20±0.76^b^	4.28±0.49^b^

Data presented are the mean ± SD. ^a^P <0.05 and ^b^P<0.01, vs. the sham group; ^c^P <0.05 and ^d^P<0.01, vs. the IR group. ABG, arterial blood gas; W/D ratio, wet/dry weight ratio of lung; IR, ischemia-reperfusion; LDH, low dose dexmedetomidine hydrochloride; HDH, high dose dexmedetomidine hydrochloride; Y+D, yohimbine and dexmedetomidine hydrochloride; Y, yohimbine.
